# Sleeping without Prescription: Management of Sleep Disorders in Children with Autism with Non-Pharmacological Interventions and Over-the-Counter Treatments

**DOI:** 10.3390/brainsci10070441

**Published:** 2020-07-11

**Authors:** Dario Esposito, Arianna Belli, Raffaele Ferri, Oliviero Bruni

**Affiliations:** 1 Child Neurology and Psychiatry Unit, Department of Human Neurosciences, Sapienza University of Rome, 00185 Rome, Italy; dario.esposito@uniroma1.it (D.E.); arianna.belli@uniroma1.it (A.B.); 2 Sleep Research Centre, Oasi Research Institute–IRCCS, 94018 Troina, Italy; rferri@oasi.en.it; 3 Department of Developmental and Social Psychology, Sapienza University of Rome, 00185 Rome, Italy

**Keywords:** autism, sleep, non-pharmacological, behavioral, complementary and alternative medicine, antihistamines, melatonin, supplement, herbal medicine, over-the-counter

## Abstract

Autism Spectrum Disorders (ASD) are lifelong neurodevelopmental conditions characterized by abnormal social interaction, communication, and behavior. Sleep disturbances represent a common comorbidity in children and adolescents with ASD, with prevalence ranging from 50 to 80%. It has been proved that sleep disruption worsens the symptoms of autism and results in challenging behaviors. Improving sleep should therefore be a primary therapeutic goal. Treatment options range from lifestyle modifications to pharmacological therapy. Several reviews have been written on pharmacological treatments, but very few on the beneficial effects of non-pharmacological interventions, over-the-counter drugs, and nutritional supplements. This study consists of a narrative review of the literature, presenting the available evidence on the following treatments: sleep education, behavioral interventions, complementary and alternative medicine (special mattresses and blankets, massage, aromatherapy, yoga, physical activity), and commonly used over-the-counter medications and supplements (antihistamines, melatonin, tryptophan, carnosine, iron, vitamins, and herbal remedies). For some treatments—such as melatonin and behavioral interventions—effectiveness in ASD is well established in the literature, while other interventions appear of benefit in clinical practice, even if specific studies in children and adolescents with ASD are lacking. Conversely, other treatments only seem to show anecdotal evidence supporting their use.

## 1. Introduction

Autism Spectrum Disorders (ASD) are a group of lifelong, heterogeneous neurodevelopmental conditions, characterized by early-onset deficits in social interaction and communication, and restricted, repetitive interests and behaviors [1].

Sleep disturbances represent a common comorbidity in children with ASD, with prevalence ranging from 50 to 80% [2]. One of the main complaints of parents of children with ASD is insomnia, which indeed is a major reason of medical consultation [3]. Sleep disturbances reported in ASD children also include parasomnias, sleep disordered breathing, sleep-related movement disorders, and excessive daytime sleepiness [4]. Emotional dysregulation, fixation on daytime events, inability to understand social cues related to sleep, anxiety, hyperarousal, and sensory processing issues are some core neurobehavioral features of ASD that may contribute to sleep disorders [5]. Furthermore, challenging behaviors may lead to ineffective or even harmful bedtime routines that are not conducive to good sleep [6]. It has been proven that sleep disruption in such children worsens the symptoms of autism [7]. Shorter sleep duration has been shown to predict greater social communication impairment, higher rates of stereotypic behaviors, and more non-functional routines [8]. Conversely, it is well established that aggressive behavior independently predicts sleep problems in children with ASD [9].

Treating these conditions should therefore be a primary therapeutic goal, given their significant impact on quality of life of patients and their families. Treatment options range from lifestyle modifications and behavioral interventions to pharmacological therapies [4]. In a stepwise approach to sleep disturbances in children with ASD—before administering prescription drugs—sleep education, behavioral interventions and over-the-counter (OTC) medications are advisable [10]. Parents generally consider behavioral interventions preferable to medication and equally effective [11]. Some non-prescription drugs—such as some antihistamines and melatonin—are among the most used substances for managing insomnia in children [12]. Complementary and Alternative Medicine (CAM) is also widely used for children with ASD [13,14], or other mental issues, given that the majority of parents perceive these therapies as a helpful and holistic therapeutic approach [15].

However, the potential of sleep hygiene and behavioral interventions is poorly understood and often underestimated by clinicians and families [16]. Moreover, some non-prescription treatments have been thoroughly studied in recent years, showing new and promising evidence for treating sleep disturbances in ASD. However, there is insufficient evidence to draw conclusions on the efficacy of other widespread remedies for sleep problems and it has been reported that only less than 20% of CAM interventions was recommended by medical doctors in the pediatric psychiatric care [13].

With regard to prescription sleep medications (e.g., α2-receptor agonists, benzodiazepines, Z-drugs, antipsychotics, and antidepressants), they should be considered to be an additional treatment option to improve sleep in children when non-pharmacological interventions are unsuccessful [17]. However, none of these sleep-promoting drugs is approved by the Food and Drug Administration (FDA) for children and adolescents with ASD. Off-label prescription in children is possible, but clinicians are often discouraged to use these molecules by their relevant potential side effects (e.g., movement disorders, hormonal and metabolic dysregulation, autonomic dysfunction).

Several reviews have been written on the pharmacological treatment of sleep disorders in autistic children [18,19,20], but few on the beneficial effects of non-pharmacological interventions, OTC treatments, and nutritional supplements. The aim of this narrative review is to evaluate the available evidence on these treatments of sleep disorders in children with ASD, to provide a useful guide to manage their sleep disorders.

We have specifically focused on the pediatric autistic population for several reasons that make this group unique and different from adults with ASD: the heavy impact of sleep disruption on neurodevelopment during infancy and childhood, the relative lack of psychotropic medications approved for the treatment of sleep disorders in pediatric age, and the distinctive therapeutic needs of patients in developmental age (e.g., parental intervention in applying sleep hygiene and behavioral techniques, and specific nutritional deficits).

## 2. Materials and Methods

This study consists of a narrative review of the literature. Extensive literature searches were conducted from 8 March 2020 to 30 April 2020 using PubMed and Web of Science core collection databases. Data for this work come from original articles, official guidelines, and previous reviews, published from the inception of databases up to 30 April 2020. The following keywords and search terms were used in different combinations: autism, autism spectrum disorder, or ASD, sleep, sleep disorders, sleep disturbances, sleep problems, insomnia, children, adolescents, behavioral sleep interventions, parent-based interventions, alternative medicine treatments, non-pharmacological treatments, alternative medicine interventions, educational program, sleep education, parent education, sleep hygiene, sport, physical activity, over-the-counter medications, non-prescription drugs, melatonin, antihistamine, supplements, vitamins, iron, tryptophan, amino acids, herbal medicine, phytotherapy. 

The reference lists of the articles retrieved through this preliminary search were examined as well, and some of them were also included in our review based on their relevance to our topic. Two authors (A.B. and D.E.) independently performed the search and screening of papers, resolving any disagreement by consensus. 

Only articles in English and Italian have been selected. We retained all types of study designs and we only excluded studies with a small sample size, given the scarcity of studies on certain topics (such as alternative medicine treatments for sleep disturbances in children with ASD). 

Whenever it was possible, we considered studies including children with ASD only, but investigations related to other populations were retained as well, if specific data on children with ASD were lacking.

## 3. Non-pharmacological Interventions

As a general tenet of pediatric practice, non-pharmacological intervention is the preferred first treatment option before initiation of pharmacologic therapies [17]. Even if sleep medications are popular treatment options for the ease of delivery (taking a pill), as well as the rapid improvement often experienced [21], they have some notable limitations, such as their short- and long-term side effects and the lack of efficacy in some disorders [22]. Accordingly, the latest guidelines for the treatment of insomnia and disrupted sleep behavior in children and adolescents with ASD, published by the American Academy of Neurology [20], recommend parent counseling to implement good sleep habits and behavioral techniques as a first-line approach. Furthermore, when there is a decision to start a pharmacologic treatment, behavioral interventions and good sleep hygiene practices should always be associated [20,23,24,25]. In other words, sleep problems in children should be approached in a stepwise fashion, with increasing intensity on a case-by-case basis when lower level strategies appear ineffective [10] (Table 1).

### 3.1. Parent-Based Sleep Education

Parent sleep education has been found to be effective in the general pediatric population [17], and in small samples of children with ASD [26,27,28,29]. A 2017 meta-synthesis of previously published systematic reviews [30] showed that parent-based sleep education programs have strong positive effects on some sleep problems of children with ASD—specifically self-settling and night awakenings—and moderate or weak positive effects on all the other sleep domains.

Nevertheless, it should be taken into account that the different possible ways of delivering sleep education may affect its outcome. A randomized controlled trial (RCT) in which parents of autistic children were given a standardized sleep education pamphlet on insomnia showed no improvement in sleep-onset latency (SOL), total sleep time (TST), wake time after sleep onset (WASO), and fragmentation of sleep [26]. Conversely, another study reported that group versus individualized sleep education did not influence the outcome in a cohort of autistic children: improvements in sleep-onset delay and other outcomes related to child and family functioning were reported regardless of the mode of education [27]. Recently, a trial was carried out to compare online and face-to-face sleep education in families of ASD children [31]. Both interventions positively impacted parent ratings of their children’s sleep behavior and parental quality of life and fatigue, even if actigraphy data showed no improvement in objective measures of sleep (i.e., TST, sleep efficiency (SE), and number of awakenings). Overall, the study demonstrated that an online parental sleep education program was as effective as a face-to-face intervention. Instead, the online program showed even better results in some areas, such as greater reduction of parental fatigue.

According to a recent review of parent training programs associated with other behavioral interventions for children with ASD [32], this kind of treatments have the potential to reduce sleep problems such as initiating and maintaining sleep, early-morning and night awakenings, and bedtime resistance.

However, more research is needed about parental education and sleep hygiene in children with ASD, especially considering that in most studies, sleep hygiene modifications were part of a multimodal intervention, including behavioral or pharmacological treatments [16,22,32,33]. The most common and evidence-based pediatric sleep practice recommendations have been reviewed [34] and recently organized in a mnemonic tool—“ABCs of SLEEPING”—[10], whose characteristics are summarized in Table 2.

### 3.2. Behavioral Interventions

Behavioral interventions include specific strategies, based on principles of learning and behavior with the aim of developing positive sleep-related habits, as well as relaxation and self-soothing skills [36].

These interventions may be classified as antecedent approaches (such as bedtime fading, stimulus fading, scheduled awakenings) and consequence-based interventions (i.e., standard and graduated extinction) [37]. The main behavioral strategies applied in the literature and in clinical practice are described below:

- Extinction. Also known as “planned ignoring”, it is the most widely studied behavioral strategy, in which the caregiver ignores all the “undesirable sleep behaviors”—such as crying—after placing the child in bed, so as to remove reinforcement for these behaviors and promote self-settling [38]. This approach (standard extinction) has been found to reduce several sleep problems among children with ASD, including night-time awakenings, co-sleeping, sleep latency, and bedtime resistance [39,40,41]. Variants of extinction are “gradual extinction” and “extinction with parental presence”. Gradual extinction differs from the standard because the parents ignore bedtime disruption just for a predetermined amount of time, before engaging with the child [40]. Gradual extinction has also been found to reduce sleep disturbances and co-sleeping; however, more research is needed on this behavioral strategy [40]. According to the strategy of extinction with parental presence, the caregiver remains in the child’s room with little or no interaction [42].
- Extinction. Also known as “planned ignoring”, it is the most widely studied behavioral strategy, in which the caregiver ignores all the “undesirable sleep behaviors”—such as crying—after placing the child in bed, so as to remove reinforcement for these behaviors and promote self-settling [38]. This approach (standard extinction) has been found to reduce several sleep problems among children with ASD, including night-time awakenings, co-sleeping, sleep latency, and bedtime resistance [39,40,41]. Variants of extinction are “gradual extinction” and “extinction with parental presence”. Gradual extinction differs from the standard because the parents ignore bedtime disruption just for a predetermined amount of time, before engaging with the child [40]. Gradual extinction has also been found to reduce sleep disturbances and co-sleeping; however, more research is needed on this behavioral strategy [40]. According to the strategy of extinction with parental presence, the caregiver remains in the child’s room with little or no interaction [42].
- Scheduled awakenings. Strategy used to reduce episodes of disorders of arousal, such as sleep terrors, which requires the child to be awoken before the usual occurrence of the sleep terror episode, in order to reduce the fearful response [37,42]. This behavioral intervention has also been proven to be effective in increasing TST [42].
- Bedtime fading. Putting children to bed before the time they effectively fall asleep (sleep-onset time) increases the chances of bedtime resistance. Therefore, in this procedure the child is initially put to bed 30 min later than his/her average sleep-onset time. Then bedtime is gradually anticipated by 30 min every few days, until reaching the desired bedtime [40].
- Stimulus fading. This strategy involves the parent gradually increasing the distance between himself/herself and the child, until he/she can fall asleep, so as to specifically reduce co-sleeping [43].
- Chronotherapy. Bedtime is gradually delayed over time, to “re-set” the circadian rhythm and improve early-morning awakenings, sleep latency and night awakenings [40].
- Bedtime “pass”. A bedtime pass is a card or any equivalent object given to the child at bedtime that may be exchanged for one “free trip out of bed” or other forms of parental consolation after bedtime. If the bedtime pass has already been used, the parent should rapidly take the child back to bed. This kind of intervention can be used to teach the child to self-soothe and stay in bed in case of difficult sleep onset or frequent night awakenings [5,18].
- Positive reinforcement. This type of intervention uses rewards to promote the desired behaviors. Rewards are typically provided for the child upon awakening in the morning [44,45,46], though sometimes reinforcements may be provided during the sleep-onset period in the form of social attention for demonstrating sleep compatible behaviors [47].
- Cognitive strategies. Group of techniques targeting the non-productive sleep-related beliefs of the child (e.g., the belief that he/she cannot improve his/her sleep), often including also coping strategies, such as teaching relaxation skills [42,48].

The successful application of behavioral approaches requires both knowledgeable psychologists or clinicians, able to teach the appropriate techniques, and motivated caregivers, who should implement these strategies consistently, despite being often challenging [17]. Therefore, it is often easier and more acceptable for parents to associate sleep education with behavioral gradual interventions (such as gradual extinction and fading), rather than abrupt approaches (e.g., standard extinction techniques) [25].

Clearly, the involvement of caregivers in the implementation of these interventions is fundamental, considering that they are usually parent-mediated [25]. Nevertheless, a recent pilot study [49] evaluated the effects of personalized behavioral sleep treatment in adolescents with high-functioning ASD, suggesting that treatment components (such as psychoeducation, relaxing bedtime routine, sleep checklist or relaxation techniques) directed towards verbal adolescents with ASD may be beneficial in addition to the more common parent-mediated sleep interventions.

Among the sleep problems addressed in the literature, behavioral interventions are the most often used to treat night awakenings, followed by bedtime disturbances [22]. On the other hand, the most common treatments employed in the literature are multi-component interventions, most often including parent psychoeducation to improve healthy sleep practices, graduated extinction, and reinforcement [22,30,42]. Notably, most of the behavioral interventions described in the literature have only minor or no difference from the techniques used with typically developing children [42].

The National Autism Center guidelines [50] considers behavioral approaches in the “emerging evidence strength” category. In particular, according to a 2017 review [30], behavioral interventions have very strong effect ranking for morning awakening, co-sleeping, and self-settling, and a moderate effect ranking on night awakenings. However, the examined papers showed high heterogeneity across study outcomes [30]. Furthermore, most of the studies evaluating behavioral interventions included only a small number of participants or showed low methodological quality, therefore limiting the ability to draw accurate conclusions about their effectiveness in children and adolescents with ASD [39,42].

Notably, among the different behavioral techniques, the choice of a specific one should be guided by the parents’ preferences [51], given that there is no conclusive evidence that one approach is more effective than another [52].

### 3.3. Other Interventions

#### 3.3.1. Weighted Blankets and Vests

Weighted blankets and vests are a widespread non-traditional treatment for sleep disturbances in children with ASD. The rational of their use is providing proprioceptive deep pressure stimulation to the child, which is postulated to result in an increased parasympathetic tone, leading to improved arousal modulation, decreased anxiety, heart rate, and cortisol, thus promoting sleep [53,54]. Despite their popularity, to the extent of our knowledge, only one study assessed the effectiveness of weighted blankets in treating sleep problems in the pediatric population with ASD. This RCT evaluated sleep with both objective and subjective methods (actigraphy and parent-reported sleep diaries); behavioral outcomes of the interventions were assessed as well. No difference was found in any sleep measure and behavioral outcome between the weighted-blanket group and the standard-blanket group. Nevertheless, the weighted blanket was favored by parents and children on subjective preference measures [55]. The above-mentioned trial found no serious adverse event associated with weighted-blanket use. Yet, in 2010 a report highlighted potential safety issues of weighted blankets and vests, following the death of an autistic child because of inappropriate use of a weighted blanket [53]. In conclusion, to date there is no evidence of efficacy and safety for the use of weighted blankets and vests as a treatment of sleep disturbances in autistic children [55].

#### 3.3.2. Sound-To-Sleep (STS) Mattress

In 2017, a preliminary randomized crossover study [56] examined efficacy and tolerability of a mattress-based technology—the STS system—in the treatment of sleep problems in 45 autistic children (aged 2.5–12.9 years). The STS mattress technology embeds sound and synched vibrations, allowing the user to both hear and feel sounds that they choose to play. The authors of this article suggested that this system may activate the parasympathetic nervous system, thus promoting sleep and relaxation. After two weeks of use, the STS system resulted in improvements in parent-reported sleep quality, and actigraphy-derived sleep duration and efficiency, with good tolerance and reported ease of use.

#### 3.3.3. Massage

The use of massage in autistic children with sleep problems is based on theoretical grounds similar to those supporting the use of weighted blankets and the above-mentioned mattress-based technologies. A systematic review evaluating non-traditional approaches to sleep problems in children with autism [54] has included the findings of four studies investigating the effect of different types of massage therapy (Qigong massage, Thai massage, and parent-provided massage). One study [57] reported the association of massage therapy with a decrease in challenging behaviors at bedtime. Another study [58] found improvements in night-time awakenings and SOL for patients receiving Qigong massage. It should be taken into account, though, that both studies included a small number of participants (20 and 15 children, respectively). A third study [59] reported an improvement in sleep behavior associated with Thai massage in a larger sample, but no specific aspect of sleep behavior was described. The fourth and last reviewed article [60] claimed an improvement of sleep behavior following Qigong massage, but sleep was measured in the context of a broader sensory assessment, making it difficult to assess whether sleep behavior specifically improved. Therefore, we can only conclude that there is weak evidence on the effects of massage therapy on sleep of autistic children.

#### 3.3.4. Aromatherapy

Only one clinical trial was carried out to evaluate the effects of aromatherapy on sleep in children with ASD [61]. During this study, aromatherapy with 2% lavender oil was administered via foot and leg massage to 12 school-aged children. SOL, sleep duration, and night awakenings where then recorded by observers who checked the children every 30 min. No difference was found in any sleep measure between the nights with and without aromatherapy suggesting no effect on sleep.

#### 3.3.5. Yoga

A recent RCT assessed the efficacy of a structured yoga intervention for sleep, gastrointestinal and behavioral problems in a cohort of 64 ASD children aged 5 to 16 years [62]. The participants were subdivided into two groups, one followed the usual school curriculum, while the other was taught yoga for 70 min/day during a three months period. A pre- and post-intervention questionnaire, including 15 questions about sleep, was completed by the caregivers. A significant improvement in sleep quality in all the investigated sleep domains was found in children following the structured yoga intervention.

#### 3.3.6. Sport and Physical Activity

Several studies examined physical activity as a variable that might influence sleep in autistic children. An observational study [63] conducted on 10 children with ASD demonstrated that activity level, as measured by actigraphy, was significantly correlated with sleep quality, as evaluated by a parent-reported questionnaire. Moreover, interventional studies were conducted to assess the impact of different physical activities on sleep quality in children with autism. Recently, an RCT [64] evaluated the effects of a basketball skill training delivered twice weekly for 12 weeks. The intervention was found to be effective in improving all sleep parameters considered (SE, SOL, sleep duration, and WASO).

A pilot study [65] with 10 participants also examined the influence of aerobic exercise and motor skills training on sleep in young patients with ASD. Children took part in thrice-weekly trainings and their sleep was both subjectively (parent-reported sleep logs and questionnaires) and objectively (sleep EEG) assessed. An improvement in SE, SOL, and WASO was found during the nights following the training. Nevertheless, such improvements were not found at the end of the 3-week period of the intervention.

In two small-sample studies, aquatic interventions have also been examined. In the first one [66], 1 h of aquatic exercise, 2 times per week, seemed to improve sleep latency and duration in 8 children aged 6–10 years. Conversely, in the second study [67], the authors found that in their sample of 40 autistic children, only those with a specific profile—high sensory sensitivity and avoidance and low autism severity—showed decreased sleep disturbance after the intervention.

In conclusion, there is mixed evidence about the effect of physical activity and specific sports on sleep disturbances in autistic children.

## 4. Oral Over-the-Counter Medicines

Sleep pills should be considered to be an additional treatment option to improve sleep in children when non-pharmacological interventions are unsuccessful [17]. However, in children with ASD pharmacological treatment is sometimes convenient as a first-line strategy together with non-pharmacological approaches, given the greater severity of sleep disturbances in these patients [25]. Both prescription hypnotic drugs and OTC sleep-promoting agents might be useful in clinical practice. Due to the scarcity of controlled studies, there is no sleep medication approved by the FDA and only one—prolonged-release melatonin for the treatment of insomnia—approved by the EMA for children and adolescents with ASD. Sleep-promoting agents are therefore widely prescribed off-label, the most commonly used off-label medications being sedating antihistamines (e.g., diphenhydramine or hydroxyzine), melatonin, α2-receptor agonists (e.g., clonidine), benzodiazepines, pyrimidine derivatives (e.g., zaleplon and zolpidem), antipsychotics (e.g., risperidone and quetiapine), and sedating antidepressants (e.g., trazodone and mirtazapine) [68].

Some of the above-mentioned agents—i.e., antihistamines and melatonin—are available over the counter in many countries all over the world. Non-prescription medications—including OTC drugs, nutritional supplements, and herbal remedies—are indeed widespread therapies for sleep disturbances in children, as they are generally easily accessible and well accepted by caregivers. Furthermore, oral non-prescription treatments are often considered to be a “safe choice” by clinicians, as they usually have less significant side effects in comparison to some prescription hypnotic drugs—such as antipsychotics and antidepressants [12,69].

Here we describe the oral non-prescription treatments and nutritional supplements most commonly used for sleep problems in children with ASD: melatonin, antihistamines, tryptophan, carnosine, vitamin D, iron, other multivitamin and mineral supplements, and herbal remedies.

### 4.1. Melatonin

Melatonin (N-acetyl-5-methoxytryptamine) is a serotonin-derived neurohormone produced by the pineal gland. Its secretion mainly happens during darkness—beginning in the evening and peaking between 2:00 and 4:00 a.m.—and is suppressed by the light [70]. Among its many biological functions (antioxidant properties, anti-inflammatory effects, involvement in early development of neurons and glia), melatonin plays a key role in establishing and regulating the circadian rhythm and has a hypnotic function, via the activation of melatonin receptors MT1 (mainly involved in REM sleep), MT2 (mainly involved in NREM sleep), or both [71,72].

Having well-known chronobiotic and sleep-inducing properties and being perceived as a “natural” substance (due to its endogenous production), melatonin is one of the most frequently prescribed drugs in children and adolescents with sleep problems [12,73,74]. More specifically, a large Australian survey reported that melatonin was prescribed in children with autism and comorbid sleep problems by 85.2% of pediatricians [12]. Initial dosages in children usually range from 1 to 3 mg/night (up to 5 mg/night in adolescents), administered 30–60 min before bedtime. If no improvements are noted, dosage can be titrated by 1–3 mg every 1–2 weeks up to a maximum dose of 10 mg. However, the clinician should consider that patients who have not responded to 6 mg/night are less likely to benefit from a higher dosage [20,75]. No serious adverse events have been associated with melatonin use. The most often reported side effects in children include headache, morning drowsiness, increased enuresis, dizziness, diarrhea, rash and hypothermia [76,77,78]. Earlier morning awakenings were sometimes reported in children supplemented with melatonin [76].

The administration of exogenous melatonin has been proven to anticipate sleep onset [79,80], without altering sleep architecture [71]. It should be taken into account, though, that the timing of administration is critical in determining the effectiveness of treatment. When used as a sleep inductor (i.e., for sleep-onset insomnia), melatonin should be administered about 30 min before the desired time of sleep [75]. By contrast, when used as a chronobiotic (i.e., for delayed sleep phase syndrome), the maximum effect on phase advance is obtained with administration 3 to 5 h before dim light melatonin onset (DLMO) for the dosage of 0.5 mg of melatonin [81]. DLMO is not predictable using sleep diaries, actigraphy, or polysomnography. However, it can be determined in home settings by measuring melatonin in saliva (collected from a cotton plug chewed for 1–2 min every hour from 7–8 p.m. to 11–12 p.m.) before starting melatonin treatment [82]. Conversely, if disrupted sleep is the main concern, melatonin treatment is usually ineffective in improving maintenance of sleep [71].

Melatonin is of special interest in ASD, in view of the abnormal central and peripheral serotonin neurobiology reported in these conditions [83,84]. In individuals with ASD, several studies reported abnormal levels of daytime or night-time melatonin, compared to typically developing controls [85,86,87,88], and nocturnal excretion in urine of 6-sulphatoxymelatonin (a melatonin metabolite) was found to correlate negatively with the severity of autistic symptoms [85]. Furthermore, children with ASD and comorbid sleep-onset delay showed genetic abnormalities in the metabolic pathway of melatonin more frequently than the general population [89].

A recent meta-synthesis [30] summarized the findings of eight systematic reviews on management of sleep disorders in children with ASD, five of which [88,90,91,92,93] included studies evaluating the effectiveness of melatonin. Two reviews included not only studies on melatonin, but also studies on other interventions for sleep disorders [90,91]. Both concluded that among the evaluated treatments, supplemental melatonin appears to have the strongest evidence supporting its use. Of the three reviews that focused on melatonin [88,92,93], one [88] was a systematic review and meta-analysis on melatonin-related findings in autistic individuals, including 18 studies on melatonin as a treatment for sleep. These studies reported improvements in SOL, night-time awakenings, and TST. Five of the studies were double-blind, placebo-controlled crossover trials and were included in a meta-analysis: significant improvements in sleep duration and latency were found, but no significant change in the number of night awakenings. Another research group [93] evaluated the effectiveness of melatonin on sleep disorders within the framework of a broader assessment of pharmacological therapies for individuals with pervasive developmental disorders, concluding that the use of melatonin may be considered for the treatment of sleep problems of these patients. Similarly, the third review [92] aimed to assess the efficacy and safety of supplemental melatonin for disordered sleep in persons with ASD. Seven studies, of the 12 identified, included children with ASD. The authors concluded that the available literature supports the use of melatonin for sleep disturbances in autistic patients, given its beneficial effects and its few and minor side effects. Finally, the overall findings of the meta-synthesis showed that melatonin had strong evidence of positive effect on SOL, TST, bedtime resistance, and co-sleeping, whereas its effectiveness was ranked as moderate in improving the longest sleep episode, night awakenings, nocturnal activity, parasomnias, sleep disordered breathing, sleep anxiety, and sleep problems not otherwise specified. Only a weak effect was found for SE, as several the studies yield negative results for this parameter [30].

Not included in the above-mentioned meta-synthesis, but of interest for its design, is a study conducted on a large sample of autistic children and adolescents over a period of 12 weeks [94]. This RCT showed that controlled-release melatonin at the dosage of 3 mg/night (1 mg immediately released and 2 mg released over a 6 h period), alone or in combination with cognitive-behavioral therapy (CBT), is effective in improving SOL, TST, WASO, and number of awakenings, as measured by actigraphy, with moderate-to-large effect sizes. Furthermore, the authors highlighted that the combination treatment group (CBT + melatonin) had higher rates of patients completing the treatment schedule and of patients obtaining clinically significant changes in sleep measures. This seems to suggest a greater effectiveness of melatonin when administered in association with a cognitive-behavioral intervention, at least in the short term.

From a practical point of view, in some children with autism a major limitation for melatonin treatment is the loss of efficacy after an initial good response [25]. Often, patients in whom the benefits of melatonin disappear after some time have been shown to have a slow metabolism of melatonin, due to a single nucleotide polymorphism in the CYP1A2 gene [95]. This may result in increased melatonin levels during daytime and, consequently, in the loss of the circadian melatonin rhythm, thus explaining the fading of effectiveness of exogenous melatonin over time [96]. As a consequence of these findings, there is now a greater understanding that low (0.5 mg), rather than high doses can work for some children, with diminishing effects for doses over 6 mg [25].

Table 3 summarizes some clinical tips on the use of melatonin, proposed during an expert consensus conference [71].

Recently, several studies have been published about the efficacy and safety of pediatric prolonged-release melatonin (PedPRM) in children with ASD. A randomized, double-blind controlled trial [97] in 125 subjects aged 2–17.5 years with ASD or Smith-Magenis syndrome showed that PedPRM mini-tablets (from 2 to 10 mg) significantly improved TST and SOL, with clinically relevant effect sizes. These improvements were well established after the first 3 weeks of treatment and then maintained throughout the entire duration of the study (13 weeks). Notably, 41% of participants responded initially to the low-dose (2 mg) PedPRM and did not require dose escalation; whereas, among the patients requiring a dose increase to the 5 mg PedPRM, half of them responded well, while the other half was eligible for further dose escalation to 10 mg. The authors did not report earlier morning awakenings in the PedPRM-treated group, differently from children treated with immediate-release melatonin [76]. There was no sign of tolerance development. Beside the decreased overall sleep disturbance, children’s externalized disruptive behaviors and caregivers’ quality of life also improved with PedPRM versus placebo [98]. The same authors then followed part of the subjects who completed the previous study up to 104 weeks of treatment, to assess long-term efficacy and safety of PedPRM [99,100]. They concluded that the improvements were maintained throughout the treatment period, and that children showed also longer uninterrupted sleep period and reduction in mid-sleep awakenings [99]. With regard to the tolerability profile, PedPRM seemed to be generally safe, the most frequent adverse reactions being fatigue (6.3%)—often reported after dose escalation and quickly resolved by decreasing the dose, sleepiness (6.3%) and mood swings (4.2%). There was no evidence of delayed growth or pubertal development and the discontinuation of PedPRM was not associated with withdrawal effects nor rebound insomnia.

### 4.2. Antihistamines

Histamine, together with other neurotransmitters (such as acetylcholine, norepinephrine, serotonin and orexin), is a major “wake-promoting” agent [101,102,103,104]. The histaminergic neurons in the central nervous system (CNS) are localized within the tuberomammillary nucleus in the posterior hypothalamus, which has therefore been described as a “wakefulness center”. These neurons project mainly to the H1 and H3 receptors of the orexin-rich perifornical hypothalamus and the cholinergic neurons in the basal forebrain. The discharge activity of this histaminergic system is highest during attentive vigilance, while it is strongly reduced during quiet wakefulness, and completely suppressed during drowsiness, NREM, and REM sleep [104]. Differently from the past, antihistamine drugs are now considered to be inverse agonists of histamine receptors, instead of pure antagonists (H1-H4) [105,106,107]. Regarding the treatment of sleep problems, the first-generation (“sedating” and non-selective) H1-antihistamines are the most commonly used agents for pediatric insomnia [69,108], thanks to their ability to pass the blood–brain barrier and their minimal effects on sleep architecture (reduced duration of REM sleep) [103,109,110]. These include ethanolamines (such as diphenhydramine) and piperazine derivatives (such as hydroxyzine), the most frequently used medications, as well as others, such as trimeprazine and niaprazine.

Not all antihistamine drugs are sold OTC in all countries, as the drug markets are differently regulated in the different nations. Nevertheless, considering that the most widely used antihistamine—diphenhydramine—is a non-prescription drug in the US, we decided to include this class of medications in our review.

The common doses recommended by manufacturers and/or by experimental studies for these drugs are summarized in Table 4.

Despite that the use of antihistamines is widespread in the pediatric population, randomized controlled studies in children are lacking [18], and this is even more true in ASD [19]. The most often used antihistamine drug, diphenhydramine hydrochloride, has generally been studied in adults [114,116]. For adults, the usual dosage ranges from 25 to 50 mg/day while in children, the recommended dosage is 0.5 mg/kg/day up to a maximum of 25 mg/day. However, only one study investigated the effectiveness of this medication in children with sleep disorders aged 2 to 12 years [111], reporting reduced SOL and night awakenings. On the contrary, a more recent RCT [112] in children younger than 2 years showed that diphenhydramine was not more effective than placebo neither in reducing awakenings nor in improving parental satisfaction of their children’s sleep. Also, hydroxyzine [113], and niaprazine [114,115] have been used to treat sleep disturbances in children: their usual dosage and effects on sleep are reported in Table 4. A randomized double-blind placebo-controlled trial [117] was conducted to test the efficacy of the antihistamine trimeprazine on sleep disturbances of infants aged 6 to 27 months, showing that this compound is clinically ineffective in treating this condition.

Other antihistamine compounds often used as sleep-inducing medications in adults include doxylamine, pyrilamine, cyproheptadine, and promethazine [107], even though they are more rarely used to treat pediatric insomnia.

As mentioned above, first-generation antihistamines cross the blood–brain barrier, therefore their most common adverse reactions at therapeutic dosage involve the CNS: fatigue, sedation, and sleepiness or, paradoxically, irritability, hyperactivity, and seizures. Notably, tolerance to these drugs can develop quickly, leading to dramatic hyperarousal as well as hyperactivity [118]. In the pediatric population, they might also affect learning: according to some authors [119], the use of a sedating antihistamine (diphenhydramine) can reduce learning abilities of atopic children if compared to second-generation (non-sedating) antihistamines or placebo. First-generation H1-antihistamines may interact also with other receptors, causing several other adverse reactions: anti-muscarinic effects (mydriasis, dry eyes, dry mouth, constipation, and urinary hesitancy and retention); anti-serotonin effects (increased appetite and weight gain); anti-alpha-adrenergic effects (dizziness and orthostatic hypotension) [109,110]. Some authors reported severe adverse reactions after the administration of diphenhydramine, including catatonic stupor, anxiety, visual hallucinations, and more rarely, respiratory insufficiency and seizures [120,121]. On the other hand, hydroxyzine is thought to be safer, with no fatal case reported [122]. An overdose of first-generation antihistamines is potentially lethal in children, due to cardiorespiratory arrest, anticholinergic syndrome (flushed skin, hallucinations, seizures, hypertension, fever), and CNS function suppression symptoms, such as lethargy, altered consciousness, and coma. Notably, infants and children often exhibit initial paradoxical hyperarousal, hallucinations, and seizures before progressing to coma [110,123].

Due to the lack of robust evidence and the relatively significant safety issues [25], antihistamines should not be used as a first-line approach for the pharmacological treatment of sleep disturbances in children and adolescents with ASD without comorbidities that would justify their use.

### 4.3. Tryptophan/5-Hydroxytryptophan

Tryptophan or L-tryptophan (Trp) is an essential amino acid, necessary for the biosynthesis of proteins. After ingestion, it participates in many metabolic pathways where it is converted into different bioactive molecules (such as serotonin, melatonin, kynurenine, and the vitamin niacin) [124]. 5-hydroxytryptophan (5-HTP) is the intermediate metabolite in the biosynthesis of serotonin from Trp and it easily crosses the blood–brain barrier. It is obtained from hydroxylation of Trp by the enzyme Trp-hydroxylase. As this is the rate-limiting step in serotonin synthesis, therapeutic doses of 5-HTP effectively increase the biosynthesis of serotonin in the CNS [25,125].

The mechanisms through which Trp/5-HTP influence sleep appear to be multiple and not completely understood. It is well established that by increasing the CNS concentration of serotonin, 5-HTP provides substrates for melatonin production and enhances serotonin-mediated regulation of sleep. The serotonergic system—in the past considered slow wave sleep-inducing but later regarded as wake-promoting—might actually have different effects on sleep that depend on its degree and timing of activation. Serotonin may indeed directly inhibit sleep, but subsequently induce a cascade of physiological processes that enhance sleep, mediated by yet to be identified sleep-promoting factors [126,127].

Trp at doses of 1 g/day or more has been shown to increase TST and decrease sleep latency and arousal after sleep onset in adults with insomnia [128,129]. It has not an opioid-like effect and does not prevent desired arousal from sleep, causing none [130] to negligible alterations of the physiological sleep architecture (minimal decrease in REM sleep and increase in NREM sleep) [131]. In subjects with neuropsychiatric syndromes and self-reported sleep disturbances, Trp given at bedtime (dosages from 1.2 g to 3 g) was found to increase the amount of slow wave sleep, without altering the amount of REM sleep [132]. Furthermore, Trp dietary intake in the general population has recently been shown to be positively associated with self-reported sleep duration [133]. As for 5-HTP, its use in healthy humans has been shown to increase REM sleep at dosages ranging from 200 to 600 mg/day [134].

Trp and 5-HTP appear to be safe and have few and mild side effects. However, in consideration of the possibility of serotonin syndrome, Trp and 5-HTP should not be used in patients currently treated with many antidepressants (such as monoamine oxidase inhibitors, SSRI, or tricyclic antidepressants) [135]. Tremor, nausea, and dizziness have been reported in association with Trp, but almost exclusively when taken at high doses (70–200 mg/kg/day) or in association with SSRI [136]. As for 5-HTP, some patients experience nausea and vomiting when taking it, especially at the beginning of treatment, but such symptoms are benign and usually transitory. It is suggested, to avoid them, to initiate the therapy at low doses and increase them gradually if necessary [135]. Reports of cases of eosinophilia-myalgia syndrome (EMS) in patients taking L-Trp in 1989 should not raise concerns about the use of Trp or 5-HTP as dietary supplements. Investigations found out that a contaminant, rather than Trp itself, had caused this illness: a new bacterial strain, used by a single manufacturer in the fermentation process of Trp, together with insufficient purification of the product, were responsible for the EMS epidemic [137]. Trp was temporarily banned from the market by the FDA, until 2005. Since then, Trp-containing products are available and commonly used as dietary supplements. After 1989, 5-HTP has become popular in place of Trp. Because of its biochemical similarity to Trp, 5-HTP has been under vigilance for its safety, but no ascertained cases of toxicity have emerged, even though 5-HTP is widely used worldwide. Extensive analyses of several sources of 5-HTP did not find toxic contaminants or any other significant impurity [137].

In the pediatric population, Trp and 5-HTP supplementation proved to be effective in reducing NREM parasomnias [138,139]. A clinical trial [139], conducted on 165 children and adolescents diagnosed with primary NREM parasomnia, compared the outcome of patients taking Trp (dose range: 500–4500 mg/day, mean dose of 2400 mg/day) with that of patients who chose not to be supplemented: an improvement in parasomnias was found in 84% of children using Trp, whereas only 47% of children not using it experienced a reduction in parasomnia symptoms (*p* < 0.001). Another clinical trial [138] proved that in a group of 45 children with sleep terrors, those taking 5-HTP (2 mg/kg/day at bedtime) not only improved their symptoms in a significantly greater proportion than those not being supplemented, but also maintained the improvements at the 6-month follow-up.

Furthermore, Trp seems to reduce night awakenings in children and adolescents. In a pilot study [140] on children aged 7 to 17 years, melatonin supplementation (“ME group”) was compared to melatonin + vitamin B6 + Trp supplementation (“MET group”): after two months of supplementation, a statistically significant reduction of the number of night awakenings was found in the “MET group”, but not in the “ME group”. Similar results were found by another study [141] conducted on a large sample of younger children (748 patients aged 12 to 48 months) that had to undergo audiologic testing requiring the child to be asleep. A solution containing melatonin, Trp and vitamin B6 proved to be significantly more effective than melatonin alone in reducing the number of tests that had to be repeated due to awakening of the child. Such findings suggest the potential benefits of Trp in improving disrupted sleep in the pediatric population.

Interestingly, morning Trp ingestion seems to also have an influence on sleep onset and termination in children. A Japanese study [142] conducted on a large cohort investigated the relationship between dietary tryptophan intake at breakfast and some sleep characteristics, subjectively assessed using questionnaires. It was found that among preschoolers, those who reported a very short SOL, also reported a significantly higher Trp intake at breakfast than those who reported a very long SOL (mean Trp intake at breakfast: 440 mg versus 302 mg). Furthermore, in the same study the amount of Trp taken at breakfast seemed to inversely correlate with the frequency of difficulties in falling asleep and in waking up.

Nevertheless, Trp appears to improve sleep in children also when administered during night-time. In a clinical trial [143] conducted on 30 infants with sleep problems, participants were given tryptophan-enriched cereals and sleep-promoting formula milk with high levels of Trp at supper. The actigraphic evaluation of their sleep showed statistically significant improvements in sleep time, time spent in the crib and SE, if compared to the same parameters recorded during a control week. Such findings, while proving the effectiveness of Trp on children’s sleep, leave uncertainties on the role played by the timing of its assumption.

As for children with ASD, experimental studies investigating the effects on sleep of Trp/5-HTP supplementation are lacking. However, data from metabolomic studies suggest alterations of the Trp metabolism in children affected by ASD [144,145,146]. More specifically, in these patients Trp appears to be preferentially transformed in metabolites of the kynurenine pathway, at the expense of the serotonin-melatonin pathway [146], thus potentially playing a crucial role in the pathogenesis of sleep disruption.

### 4.4. Carnosine

Carnosine or L-carnosine is a dipeptide composed by alanine and histidine, which acts as an antitoxic and neuroprotective agent in the nervous system and muscles [147]. Its circulating levels have been shown to be lower in autistic patients than controls [148,149] and recent studies suggest that high oxidative stress might contribute to the worsening of sleep disorders and behavioral symptoms in ASD individuals [88,150]. Some authors [151], therefore, suggested that 800 mg/day of carnosine might improve communication and behavior of autistic children.

As a consequence of this background, in a recent controlled trial [152], the effects of carnosine supplementation on children with ASD were tested. A cohort of 43 children with ASD, aged between 4 and 16 years, was randomized to 500 mg of L-carnosine or placebo, administered once daily for 2 months. The authors then monitored the effects of this treatment on sleep parameters and core autistic symptoms, through parent-report questionnaires. At the end of the supplementation period, significant improvements were observed in sleep duration, parasomnias, and total sleep disorders scores in the carnosine group, even though no effect size was provided. On the other hand, in disagreement with the previously cited study [151], the supplementation was found to have no effect on autism severity. No significant adverse reaction was reported by these authors.

### 4.5. Iron

Iron is the most abundant metal in the CNS and is crucial for various metabolic pathways within the brain. Its homeostasis in cerebral cells is therefore accurately regulated, so that the brain is less affected by acute diseases that reduce peripheral iron levels (e.g., acute anemia) in comparison with other organs. However, chronic imbalance of iron homeostasis can affect several cellular processes in which iron is involved: catecholamine synthesis and reuptake, serotonin synthesis, affinity, and expression of dopaminergic D2 receptors, mitochondrial efficiency, and myelination of neurons [153]. Furthermore, it has been shown [154] that iron deficiency—especially in the early stages of brain development—can lead to structural brain damages of several regions, such as hippocampus, basal nuclei, and cerebellum.

In recent years, the association between iron deficiency and sleep disorders has received growing attention in the scientific literature [155]. Low levels of iron have been associated with restless legs syndrome/periodic limb movement disorder (RLS/PLMD) [156] and RLS-induced insomnia [157,158], parasomnias [159], sleep disordered breathing [160], attention deficit and hyperactivity disorder (ADHD) [161], and “restless sleep disorder”, a newly proposed diagnosis [162].

The prevalence of iron deficiency peaks in children [163], and even more in those with ASD. This might be also due to their frequently narrow food preference, which could lead to inadequate intake of iron and substances modulating its bioavailability (“enhancers”, such as vitamin C or citrate; or “inhibitors”, such as calcium and fibers) [164,165,166]. During the first stages of iron deficiency, ferritin levels—reflecting the iron stores—drop faster, while hemoglobin remains normal. As a consequence, measuring serum ferritin is suggested to detect non-anemic iron deficiency, especially in children with ASD and sleep disorders [18,167,168], even if false increases in ferritin levels are possible because ferritin is an acute-phase reactant [169].

However, it should be noted that data correlating ferritin concentration with sleep parameters in autistic children are unclear and further research is needed. Some authors [170] described in a group of 102 children (68 with ASD, 16 with developmental delay, and 18 controls) an association between low serum ferritin levels and sleep fragmentation with reduced SE, but a cause/effect relationship could not be confirmed. Moreover, in a retrospective study [171] enrolling 53 children with ASD, it was found that serum ferritin was significantly lower in patients with ASD and poor SE, compared to those with good SE, as measured by polysomnography. This study also reported that PLMD was significantly more frequent in children with ASD than in controls, but there was no significant difference in ferritin levels between autistic patients with and without PLMD. In another study [172], the effect of iron supplementation in 33 children with ASD and restless sleep was investigated. After 8 weeks of treatment with 6 mg/kg/day of elemental iron, the children’s restlessness in bed improved significantly, as well as mean ferritin levels (from 16 μg/L to 29 μg/L), hemoglobin, and mean corpuscular volume. However, no correlation was found between restless sleep scores—as measured by two sleep questionnaires—and serum ferritin concentration. No improvement was described in other sleep parameters either.

As has been shown, only limited data are currently available for children with ASD. Nevertheless, many authors suggest the administration of iron supplements in this population when low ferritin levels (below 30–50 ng/mL) and poor sleep quality are both present [20,25,164]. The recommended initial dose in the pediatric age is usually 1–2 mg/kg/day of elemental iron divided in 2–3 doses between meals; the clinician may then titrate up to 6 mg/kg/day, in order to reach a serum ferritin concentration of 30–50 ng/mL or more [20,173]. Follow-up visits should be scheduled within 2–3 months of oral iron initiation, to check ferritin levels and to identify potential hinders to iron absorption and adherence to therapy [167]. Notably, the elemental iron should not be administered with milk or dairy products, nor with anti-acids, H2-receptors antagonists, and proton-pump inhibitors, which can affect iron absorption [166]. Compliance to therapy—especially in the pediatric age—has been shown to be the main problem for iron supplementation in children with RLS [167]. Indeed, many factors can affect adherence to treatment: poor palatability, frequent gastrointestinal side effects—such as constipation—[20,174], difficulty in swallowing pills, or teeth staining for liquid preparations [175].

### 4.6. Vitamin D

In healthy individuals, vitamin D, a fat-soluble pro-hormone, is mainly obtained by endogenous production taking place in the skin through the action of B band ultraviolet (UVB) solar radiation on a precursor molecule. A smaller amount of vitamin D also comes from dietary intake and supplementation. Vitamin D is then hydroxylated to 25-hydroxyvitamin D (25(OH)D) in the liver, and subsequently converted into the metabolically active form 1,25-dihydroxyvitamin D (calcitriol), primarily in the kidneys [176]. Maintaining calcium homeostasis and bone health are traditionally known as the main roles of vitamin D; however, there is growing evidence that vitamin D contributes to sleep regulation too and two mechanism have been proposed. First, similarly to other neuroactive steroids, vitamin D can modulate neuronal excitability via its receptors (VDR) in the brain. Several studies reported vitamin D to bind VDR in many sleep-regulating areas of the CNS (such as the anterior and posterior hypothalamus), thus potentially modulating their activation. Second, it has been found that the tryptophan hydroxylase-2 (TPH2) gene is regulated by a sequence of DNA known as the vitamin D response element. Vitamin D can therefore induce the expression of this brain enzyme and, consequently, the production of 5-HTP, precursor of serotonin and melatonin [177]. Furthermore, vitamin D has also been linked to the pathophysiology of RLS [178] through two main mechanisms: dopaminergic dysfunction and iron dysregulation. VDR are indeed highly expressed in the midbrain, in particular in the substantia nigra, supporting dopaminergic neurons survival by increasing glutathione and reducing oxidative stress in experimental models [179,180,181]. Moreover, it has been demonstrated that low vitamin D levels are often associated with iron deficiency in children, even if there is no clear etiological explanation for this correlation [182].

A prospective study on a large French pediatric cohort suggested an association between low serum levels of 25(OH)D in the cord blood and an increased risk of being a persistent short sleeper in preschool age [183]. However, another observational study [184] did not confirm the association between cord blood vitamin D deficiency and any sleep parameter, neither objectively nor subjectively assessed, at the age of 2 years. Nevertheless, the same study found that children with vitamin D deficiency at the age of 2 years had significantly shorter night sleep duration and total sleep duration—measured both subjectively and objectively—than those with normal vitamin D levels (*p* < 0.05). Furthermore, a clinical trial conducted in older Chinese children (age range 8–14 years) showed that 25(OH)D serum levels are positively correlated with sleep duration and that insufficiency/deficiency of vitamin D (25(OH)D ≤ 20 ng/mL) is independently associated with an increased probability of insufficient sleep [185].

In children with ASD—to our knowledge—no adequate interventional study has been carried out to investigate the effects of vitamin D supplementation on sleep. Despite that, in recent years an increasing number of studies have shown lower serum levels of vitamin D in children with ASD than in neurotypical children [186,187]. Consequently, it might be useful to assess vitamin D levels in autistic children with sleep disturbances [18]. A fairly large consensus exists that when a vitamin D deficiency is found, an appropriate dosage for supplementation in all infants and most children is 400–600 international units (IU)/day, i.e., approximately 10–15 μg/day. [188]. However, some authors reported that vitamin D blood levels are more relevant than the administered dose in improving sleep quality, with the most beneficial effects on sleep being obtained for 25(OH)D hematic concentrations of 60–80 ng/mL [189].

### 4.7. Multivitamin and Mineral Supplements

Based on the finding that plasma levels of many vitamins and minerals are often lower in children with ASD than in neurotypical children, a placebo-controlled trial was conducted in which multivitamin and mineral supplements were administered for 3 months to autistic children, aged 3 to 8 years [190]. At the end of the study period, many domains of health and behavior of the children, including sleep, were assessed by means of a parent-report questionnaire. Compared to the placebo group, the group taking the supplement had slightly better scores in the sleep domain of the questionnaire (*p* < 0.05). However, the small sample size and the lack of detailed sleep assessment before and after supplementation make the evidence provided by this study quite weak. A similar study was conducted later on a larger group of children and adults with ASD [191], but the sleep scores of the questionnaire showed no statistically significant difference between the placebo group and multivitamin and mineral supplement group. These findings suggest that to date, there is no evidence supporting the use of multivitamin and mineral supplements for the treatment of sleep disturbances in autistic children and adolescents.

### 4.8. Herbal Remedies

The use of herbal remedies as an alternative treatment for sleep disturbances is widespread because these products are readily accessible over the counter and generally perceived to be safe [192]. However, there is limited scientific support of the efficacy and safety of many herbs traditionally known for their sedative effects [125,193]. We will here review the evidence available for valerian, lemon balm, passionflower, lavender, St. John’s Wort, and chamomile.

Valerian (*Valeriana* spp.) is the most studied herbal remedy for insomnia. Extracts from its roots are commonly used for their sedative and anxiolytic effects and it has indeed been shown that they may modulate cortical excitability in humans [194]. As for other herbal remedies, the therapeutic effects of valerian may rise both from the individual effects of each of its constituents (volatile sesquiterpenes, valepotriates and amino acids) and from their reciprocal interactions on the gamma aminobutyric acid (GABA) and adenosine systems [195,196]. As a sedative, valerian is usually administered 30 to 60 min before bedtime, with an average daily dose of 912 mg (ranging from 300 to 3645 mg/day in different studies) [125,197]. Results of clinical trials testing the effects on sleep of different species of valerian (*officinalis*, *edulis*, *wallichii* or unspecified) are conflicting and controversial. In 2015, a meta-analysis [197] including 12 RCT described no significant difference between valerian supplements and placebo in improving SOL, sleep duration, SE, WASO, or sleep quality. Nevertheless, a recent systematic review [193] including 17 studies—nine of which did not appear in the meta-analysis cited above—reported mixed findings: several trials showed improvements in sleep quality and other subjective parameters of sleep after administration of valerian [198,199,200], while other studies did not find any significant effect [201,202,203]. However, many of these trials had relevant limitations, such as the lack of objective measures of sleep parameters or small samples. Regarding adverse effects of valerian, gastrointestinal upset, contact allergies, headache, restless sleep, and mydriasis have rarely been reported [125]. One study [204] signaled a significantly greater incidence of diarrhea in subjects treated with valerian; however, this has not been confirmed by other studies [197].

Lemon balm (*Melissa officinalis*) is a perennial herb alleged to have hypnotic properties [205]. In healthy volunteers, the administration of 240 mg/day of *Melissa officinalis* extract, together with 360 mg of *Valeriana officinalis* root extract, before bedtime, improved sleep quality with a statistically significant difference over placebo [206]. In patients with mild-to-moderate anxiety and sleep disturbances, 600 mg of lemon balm leaf extract were found to reduce anxiety-related insomnia [207] and in menopausal women 160 mg of *Melissa officinalis* extract, associated with 320 mg/day of *Valeriana officinalis*, reduced symptoms of sleep disorders [200]. The effectiveness and tolerability of a combined valerian/lemon balm preparation on dyssomnia were investigated in children as well [208]. In this study, conducted on a large sample of patients younger than 12 years, dosages were patient-tailored, up to 640 mg/day of valerian and 320 mg/day of lemon balm, and the treatment lasted for 4 weeks (+/−1 week). In total, 80.9% of the patients who suffered from dyssomnia experienced an improvement and the supplement was judged to have a good tolerability in 96.7% of them. However, it should be noticed that symptoms were subjectively assessed by parents without a standardized and validated questionnaire and that the study was not placebo-controlled. Another clinical trial, aiming to assess the action of different herbal preparations on parent-reported sleep bruxism in children, reported mixed findings: Melissa officinalis resulted effective when symptoms were assessed through a Visual Analogue Scale, but ineffective, when the sleep diaries were considered [209].

*Passiflora incarnata*, commonly known as passionflower, has a history of use for the treatment of anxiety and insomnia symptoms [210]. An experimental study showed that passionflower can positively affect sleep regulation by modulating the expression of several circadian clock genes [211]. However, its exact mechanism of action is not entirely known. A recent RCT investigated the effects of passionflower extract tea on polysomnographic sleep parameters in a large sample of adults with insomnia, finding a significant increase in TST—but not in SE and WASO—in the passionflower group compared to the placebo group [212].

Lavender (*Lavandula*) has been studied in recent years for its effects on anxiety and sleep in adults. In 2010, in a RCT [213], 80 mg of oral lavender oil supplements (silexan) administered for 6 weeks were shown to be as effective as a common benzodiazepine (lorazepam) in adults with generalized anxiety disorder, improving both anxiety scores and subjectively reported sleep quality. Moreover, two other RCT [214,215] confirmed the anxiolytic effect of 80 mg/day of silexan for 10 weeks, also suggesting its effectiveness in reducing anxiety-related disturbed sleep, as measured by the Pittsburgh Sleep Quality Index (PSQI). Finally, in 2012 a phase II open-label trial on patients with post-traumatic stress disorder or somatization disorder [216], showed an improvement in morning tiredness and night awakenings, as reported in their sleep diaries, after 6 weeks of treatment with 80 mg/day of silexan. The main adverse reactions reported were mild or moderate gastrointestinal complaints.

St. John’s Wort (*Hypericum perforatum*) is a flowering herb used for a variety of clinical conditions, such as depression, anxiety, and sleep disorders. The most active components are thought to be hyperforin and hypericin, although other components have bioactivity [125]. A major role is apparently held by hyperforin, which inhibits the reuptake of serotonin, norepinephrine, dopamine, GABA, and L-glutamate [217,218], even if further research is needed to clarify the mechanisms of action of *Hypericum* [219]. No published clinical trial specifically investigated the effects of St. John’s Wort on sleep disturbances. However, it has been reported that in adults, 900 mg of St. John’s Wort decreased scores of the sleep problem scale [220] and increased REM sleep latency [221] compared with placebo. Side effects associated with St. John’s Wort during clinical trials included gastrointestinal complaints, dizziness, fatigue, anxiety, photosensitivity and headaches [125]. However, the main concern about St. John’s Wort use is the possibility of severe drug interactions, mediated by the CYP450 and intestinal P-glycoprotein induction and by its serotonin-promoting effects.

German chamomile (*Matricaria recutita*) is a member of the daisy plant family, widely used as a natural remedy for a variety of mild clinical problems, among which insomnia. Sedative effects of German chamomile are mild and may be due to a benzodiazepine-like compound found in the flowerhead, the flavonoid apigenin, which binds the benzodiazepine receptors [192]. Although chamomile has a popular reputation of being a relaxing and sleep-facilitating compound, clinical studies are scarce. A study on elderly people who were administered chamomile extract (400 mg/day) for 28 days reported improvements in general sleep quality and SOL assessed by the PSQI [222], while another study on sleep-disturbed postpartum women found a mild improvement only in self-reported “physical symptoms-related sleep inefficiency” after 2 weeks of chamomile tea, but not after 4 weeks [223]. Moreover, in a randomized, double-blind, placebo-controlled pilot trial with subjects suffering from primary chronic insomnia, no significant differences over placebo were found in Insomnia Severity Index (ISI) and PSQI following the administration of 540 mg/day of chamomile for 28 days [224].

To the extent of our knowledge, no study has evaluated the clinical effectiveness of the above-mentioned herbal remedies on sleep disturbances in children with ASD [54].

## 5. Conclusions

Sleep disturbances in children with ASD are a challenging issue for patients, families, and clinicians. Non-pharmacological and oral non-prescription treatments are widely used to treat these conditions. Despite that, the available evidence on the subject is limited and often controversial. We reviewed the literature concerning these therapeutic approaches, to provide practitioners with a comprehensive guide.

The first step in approaching sleep problems in children and adolescents with ASD should always be parent counseling to implement good sleep habits and behavioral techniques. We have reported above the mnemonic tool “ABCs of SLEEPING” summarizing several evidence-based sleep hygiene practices, and the most studied behavioral interventions for sleep in children, such as standard and gradual extinction, positive reinforcement, and bedtime fading. Notably, the behavioral approach to use should be chosen according to the parents’ preferences, given that there is no conclusive evidence that one technique is more effective than another, and that caregivers motivation is crucial to the success of these interventions. Furthermore, when there is a decision to start a pharmacologic treatment, behavioral interventions and good sleep hygiene practices should be always maintained.

With regard to the other non-pharmacological interventions reviewed (weighted blankets and vests, mattress-based technologies, massage, aromatherapy, yoga, sports, and physical activity), no strong evidence recommending their use to improve sleep in children and adolescents with ASD is available. However, promising results come from a small number of clinical trials using mattress-based technologies, yoga therapy, massage, and physical activity, showing at the same time no significant adverse effect. While awaiting further well-designed studies on larger samples, some of these interventions might be reasonable options in specific cases, even though we recommend that clinicians inform parents of the lack of strong evidence, if asked for their opinion on these interventions.

Among the oral non-prescription sleep-promoting agents reviewed above, melatonin is one of the most used and studied substances in children with ASD, given their often-abnormal melatonin plasma levels. Melatonin has been shown to be both effective and relatively safe and has, indeed, strong evidence of positive effect on SOL, TST, bedtime resistance, and co-sleeping, while it has only moderate or lower effects in improving other sleep problems, such as frequent night awakenings. Furthermore, a greater effectiveness of melatonin has also been suggested when administered in association with a cognitive-behavioral intervention. Recently, a prolonged-release formulation of melatonin has been approved for use in children with ASD. Advantages of this treatment compared to immediate-release melatonin are improvements in sleep disruption, night-time and early-morning awakenings, as well as in disruptive behaviors. In long-term studies (up to 104 weeks) no significant adverse event has been reported, excluding also relevant consequences on pubertal growth.

As for sedating antihistamines, despite their widespread use as sleep-inducing agents in the pediatric population, there is limited evidence of their effectiveness in improving sleep in children, and no evidence at all for children and adolescents with ASD. Moreover, the frequent and quickly developing tolerance to these molecules, as well as their relatively significant safety issues, make antihistamines unsuitable medications for a first-line approach to sleep disturbances in autistic children and adolescents. Nevertheless, their use may be appropriate in patients that do not benefit from first-line treatment strategies, as well as in those with comorbid allergic symptoms.

Regarding tryptophan and its metabolite 5-HTP, the alterations of their metabolism found in patients with autism make these molecules a reasonable treatment option for autistic children with comorbid sleep problems, especially for those experiencing parasomnias and frequent night awakenings; tryptophan and 5-HTP have indeed been found to improve such symptoms in neurotypical children. Other positive aspects of this supplement seem to be the relative absence of side effects and the lack of development of tolerance with long-term use.

As for carnosine, it has shown promising, yet preliminary results in improving sleep in a small sample of autistic children and adolescents; further research is needed to confirm the effectiveness of this molecule as a treatment for sleep disorders in children with and without ASD.

We also reviewed the increasing evidence linking iron deficiency to sleep disorders: even if this topic has been thoroughly investigated in the pediatric age, the data currently available on children with ASD are scarce. Nevertheless, some authors suggest checking serum ferritin levels in autistic children with sleep disturbances and to administer oral iron supplements when low ferritin levels and poor sleep quality—especially restlessness in sleep—are both present.

As for vitamin D, even if no clinical trial has proven beneficial effects of this molecule on sleep in the pediatric population with ASD, there is sufficient evidence that vitamin D deficiency in children is associated with bad sleep. It is, therefore, often suggested to monitor serum 25(OH)D level in all children and adolescents with ASD and comorbid sleep disorder and to supplement them if vitamin D deficiency is found.

Tentative treatments with multivitamin and mineral supplements for sleep problems have also been carried out, based on the finding of low plasma levels of many vitamins and minerals in children with ASD, but the available evidence does not support this kind of treatment.

Concerning herbal remedies, to the extent of our knowledge, no study has evaluated the clinical effectiveness of valerian, lemon balm, passionflower, lavender, St. John’s Wort, and chamomile on sleep disturbances in children with ASD. Moreover, even in other populations the evidence is limited and often mixed. Promising results in improving sleep have been found by some studies on adults and neurotypical children using valerian—alone or in association with lemon balm-, passionflower, and lavender oil. Nevertheless, well-designed, placebo-controlled clinical trials on larger samples are needed to confirm such findings. Therefore, the use of these substances—alone or as an adjunctive therapy—in the autistic pediatric population should be guided by clinical experience and parents should be provided with information about their known efficacy, safety profile, and possible interactions with other treatments—especially for St. John’s Wort, the use of which should be discouraged, given the relevant safety concerns.

In conclusion, whereas for some non-pharmacological interventions and oral non-prescription treatments effectiveness on sleep disturbances in ASD children and adolescents has been ascertained, for other remedies—despite their common use—further and rigorous research is needed.

## Figures and Tables

**Table 1:** Stepwise approach to sleep disturbances in children. Adapted from Howlett et al. (2020) [10].

**Level 1**	Sleep education and implementation of healthy sleep practices
**Level 2**	Specific behavioral strategies
**Level 3**	Medications

**Table 2:** Evidence-based pediatric sleep practice recommendations of the ‘ABCs of SLEEPING’ mnemonic. Adapted from Howlett et al. (2020) and Allen et al. (2016) [10,34].

Practice Areas	Recommendations	Level of Empirical Support ^1^
Age-appropriate Bedtime, wake-times, and naps with Consistency	- Consistency of bedtimes, wake-times, and naps	Strong
	- Bedtime no later than 9 p.m.	Moderate
	- Age-appropriate amount of sleep ^2^	Moderate
Schedules and routines	- Consistent and relaxing bedtime routine	Strong
	- Daytime activities should not interfere with sleep	Moderate
Location	- Dark, cool, and quiet sleep location; limit distractions	Limited
	- Activities in bedroom restricted to quiet play and sleep	Limited
No Electronics in the bedroom or before bed	- No electronics in bedroom	Strong
	- Do not use electronics within a 1 h before bedtime	Limited
Exercise and diet	- Physical activity on a daily basis	Equivocal
	- Well-balanced and healthy diet	Limited
Positivity and relaxation	- Positive living atmosphere, free of conflict	Moderate
	- Child should feel relaxed and calm before bed	Limited
Independence falling asleep	- Encourage falling asleep and staying asleep without parent help	Strong
Needs met during the day… equals Great sleep	- Satisfy emotional and physiological needs during daytime	Moderate

^1^ levels of empirical support as defined by Allen et al. (2016) [34]. ^2^ see Paruthi [35].

**Table 3:** Recommendations for prescribing melatonin in children with ASD. Adapted from Bruni et al. (2015) [71].

Consider melatonin in children with	Sleep-onset insomnia and/or difficulty awakening up in the morning.
Measure…	DLMO when possible before starting the treatment.
Minimum age for administration	Administration after 6 months of age is generally considered safe.
Time of administration in children	- If used as sleep inductor: 30 min before bedtime.
	- If used as chronobiotic: 2–3 h before DLMO or 3–4 h before actual sleep-onset time.
Dosage	If used as a sleep inductor:
	- start with 1–3 mg (up to 5 mg in adolescents);
	- if needed, increase by 1–3 mg every 1–2 week until effect (maximum 3 mg if < 40 kg; up to 5–6 mg if > 40 kg (see text)).
	If used as chronobiotic:
	- start with a low dose of 0.2–0.5 mg;
	- if needed, increase by 0.2–0.5 mg every week until effect;
	- if no response after 1 week: increase dose by 1 mg every week until effect;
	- when 1 mg is effective: try lower dose.
Treatment duration	- Treatment duration should be tailored to the specific patient but in general should not be less than 1 month;
	Stopping successful treatment too early may result in relapse of insomnia.
	- Treatment can be withdrawn just before puberty (around 12 years of age) or shortly thereafter.
	- Stop melatonin treatment once a year for one week (preferably in summer) after a normal sleep cycle is established.
When melatonin treatment is not effective/loses effectiveness over time	- Check timing of administration.
	- Be aware that loss of efficacy of melatonin treatment is most likely caused by slow melatonin metabolism: in some cases, dose reduction is warranted instead of dose escalation.
	- Reconsider diagnosis: look for neuropsychiatric and medical comorbidities and treat them.
	- Concomitant medications can influence melatonin metabolism:
	e.g., ciprofloxacin, cimetidine, fluvoxamine (inhibitors) or carbamazepine, insulin, omeprazole (inducers).
If sleep maintenance problems after start of melatonin treatment	Melatonin dose is probably too high.

DLMO = Dim Light Melatonin Onset.

**Table 4:** Recommended dosages of some commonly used antihistamines for treating sleep disorders in children.

Antihistamine	Dosage	Effects on Sleep	Refs
Diphenhydramine	0.5 mg/kg up to 25 mg/day	↓ SOL; ↓ arousal threshold	[111,112]
Hydroxyzine	0.5–1 mg/kg/day	↓ SOL; ↓ arousal threshold	[113]
Niaprazine	1 mg/kg/day	↓ SOL; ↓ arousal threshold; effect on sleep maintenance (e.g., ↑ TST)	[114,115]
**Most common side effects**: daytime drowsiness, gastrointestinal disturbances (vomiting, constipation), paradoxical excitation, anti-cholinergic effects [25].			

↓ = decrease; ↑ = increase.
